# Subgap Absorption in Organic Semiconductors

**DOI:** 10.1021/acs.jpclett.3c00021

**Published:** 2023-03-24

**Authors:** Nasim Zarrabi, Oskar J. Sandberg, Paul Meredith, Ardalan Armin

**Affiliations:** † Sustainable Advanced Materials (Ser-SAM), Department of Physics, Swansea University, Singleton Park, Swansea SA2 8PP, United Kingdom

## Abstract

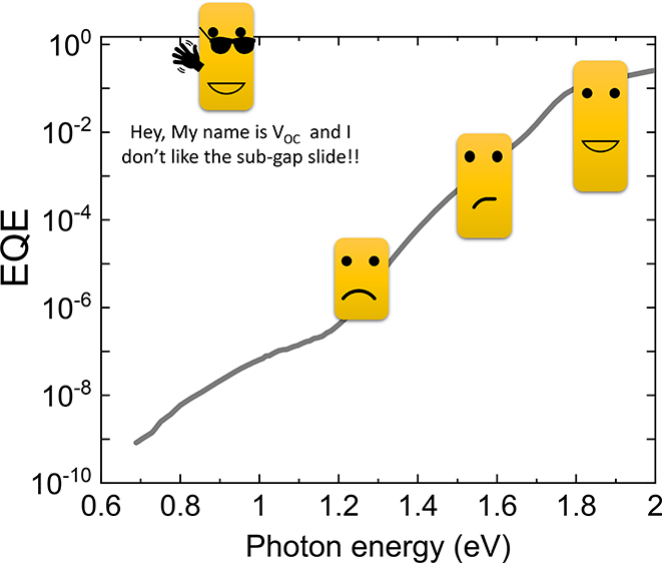

Organic semiconductors have found a broad range of application in areas such as light emission, photovoltaics, and optoelectronics. The active components in such devices are based on molecular and polymeric organic semiconductors, where the density of states is generally determined by the disordered nature of the molecular solid rather than energy bands. Inevitably, there exist states within the energy gap which may include tail states, deep traps caused by unavoidable impurities and defects, as well as intermolecular states due to (radiative) charge transfer states. In this Perspective, we first summarize methods to determine the absorption features due to the subgap states. We then explain how subgap states can be parametrized based upon the subgap spectral line shapes. We finally describe the role of subgap states in the performance metrics of organic semiconductor devices from a thermodynamic viewpoint.

Ideally, the band structure of a highly ordered inorganic semiconductor, in its generic definition, includes a forbidden gap within which no electronic state is allowed. This results in the absence of light absorption at photon energies below the bandgap, the so-called subgap region. In the presence of inevitable defects and imperfections, however, disorder-induced tail states located within the bandgap have been observed to absorb light in the subgap region.^1^ The spectral line shapes due to these states provide information about their energy, distribution, and nature. Even in a (thermodynamically impossible) perfectly ordered crystal, subgap light absorption is present due to the dynamical disorder induced by lattice phonons.^2^ In disordered semiconductors such as amorphous silicon (a-Si), structural disorder results in additional broadening of the absorption onset and more pronounced subgap absorption than in crystalline solids.^3−5^

In organic semiconductors, the above-gap light absorption and the associated energy gap are determined by singlet excitons, composed of strongly bound electron–hole pairs. In addition, although there may be some degree of crystallinity, these semiconductors are predominantly disordered, and therefore, the subgap absorption is significant even in their purest form.^6−8^ The purity of organic semiconductors is mainly limited by the boundaries of the purification methods of wet chemistry. As such, the presence of extrinsic impurities and defect states are to be expected, which may also contribute to the overall subgap absorption.^9^ Additionally, in organic semiconductor blends most notably used in organic solar cells and photodiodes, the subgap absorption is typically dominated by partially radiative intermolecular charge transfer (CT) states.^10−14^ However, the absorption coefficients associated with CT states and impurities are generally several orders of magnitude smaller than those of singlet excitons.

The absorption coefficient, α(*E*), is a measure of the transition rates between electronic states in atomic and molecular spectroscopy (as a function of photon energy *E*). Depending on the energetics of the electronic states, their density, and the oscillator strength of the transitions (upon light absorption), the spectral fingerprints appear at different energies and intensities. Therefore, absorption coefficient measurements have widely been used to extract information about electronic states in semiconductor material systems. This is of particular importance in photovoltaic semiconductor devices, such as solar cells and photodetectors, where the performance is governed by the bandgap energy of the semiconductor in use and can be strongly influenced by energy states within the bandgap.^13^ In the case of organic donor/acceptor blend photovoltaic devices, subgap absorption plays a pivotal role in defining photovoltage losses and, ultimately, the losses in the power conversion efficiency, while the spectral line shape of the absorption coefficient has been widely used to infer information about the static disorder,^15^ Urbach energy tail states,^16−18^ CT states,^10,19−23^ and trap states.^24−26^

In this Perspective, we review different methods used to determine subgap absorption in organic semiconductors and explain how subgap spectral line shapes can be interpreted to obtain information about the subgap states. Furthermore, the most recent empirical findings and progress on the role that subgap states play in determining the performance metrics of organic solar cells will be discussed through their relation to the photovoltaic external quantum efficiency (EQE).

## Experimental Methods for Measuring Subgap Absorption

For a thin film of an organic semiconductor, the absorption coefficient above the energy level gap (*E* > *E*_gap_) can be inferred from the absorptance obtained from transmission and reflection (*T* and *R*) measurements (known as UV–vis spectroscopy) or ellipsometry spectroscopy.^27^ These measurements are typically limited to dynamic ranges of 20 dB (corresponding to 2 orders of magnitude) or lower due to limitations of transmission spectroscopy and parasitic Fresnel reflection from thin-film surfaces. In practice, this means that α(*E*) lower than 1000 cm^–1^ cannot be determined for typical thin-film thicknesses of 100 nm. Note that the absorptance (*A*) is the ratio between the absorbed and the incident light power, which for thin films can be approximated as *A* = *αd*.

Due to the small absorption cross section of the subgap energy states (*E* < *E*_gap_), which are the focus of this perspective, the corresponding α(*E*) is often several orders of magnitude lower than 1000 cm^–1^. As a result, more sensitive measurement techniques with considerably higher dynamic ranges are required for determination of α(*E*) in the subgap energy region. For this purpose, both optical and electrical measurement techniques have been utilized. Three measurement techniques are typically employed for the subgap absorption measurement: photothermal deflection spectroscopy, Fourier transform photocurrent spectroscopy, and sensitive external quantum efficiency.

**Figure 1:**
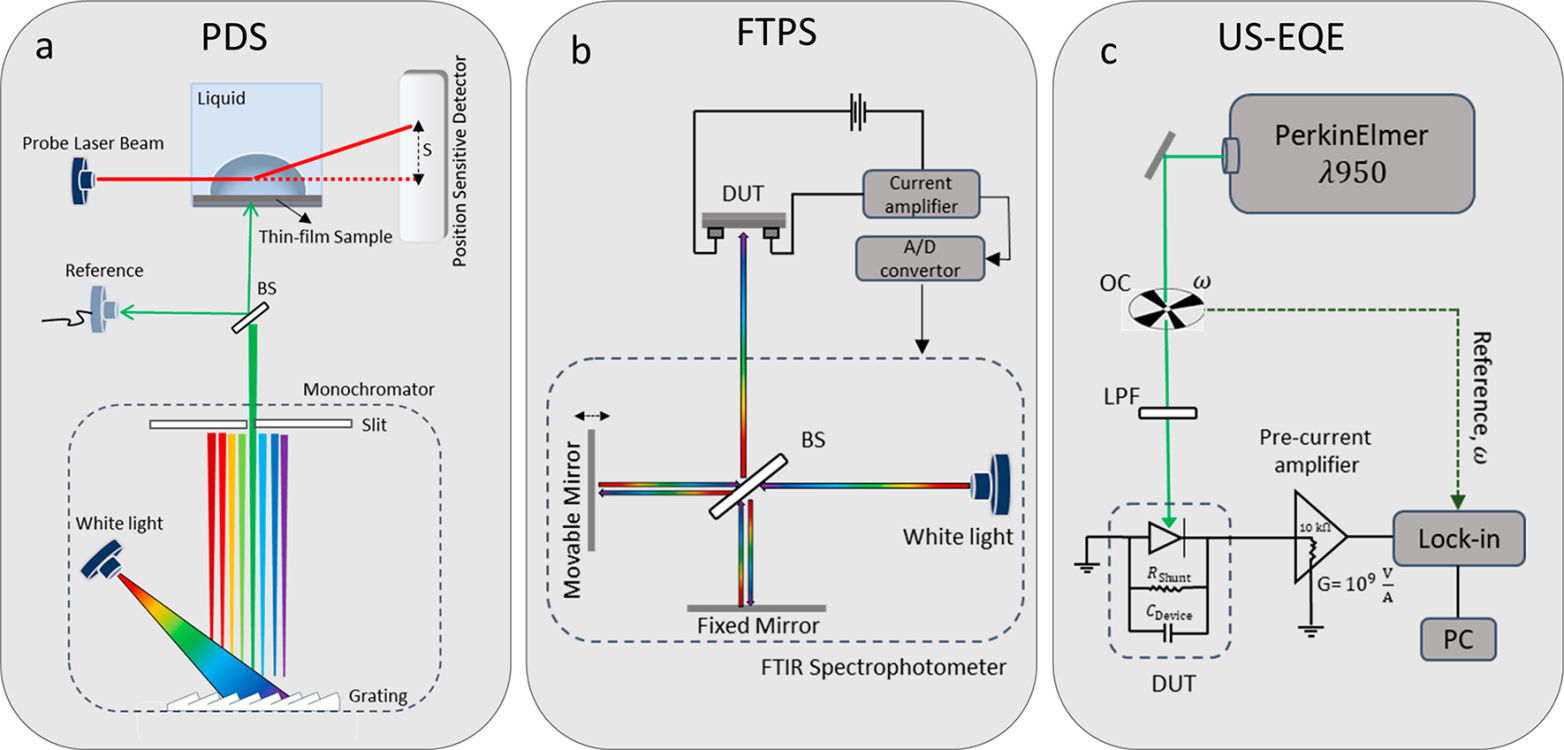
Standard experimental methods for measuring subgap absorption: (a) photothermal deflection spectroscopy (PDS); (b) Fourier transform photocurrent spectroscopy (FTPS); and (c) ultrasensitive external quantum efficiency (US-EQE).

Photothermal deflection spectroscopy (PDS) is an optical method (see Figure 1a) used to determine α(*E*) in thin-film semiconductors in the weak absorption limit (*αd* ≪ 1).^28^ This can be either for absorptance measurements of ultrathin film thicknesses in the above-gap region or for spectral regions where absorption cross section and/or oscillator strength are small (subgap regions). In this method, the thin-film sample is placed in a cuvette filled with a liquid whose refractive index is highly dependent on the temperature (*T*). For this purpose, carbon tetrachloride with  is often used, where *n* is the reflective index. The sample is then pumped by monochromatic light. As a result of light absorption, the sample is heated up. A temperature gradient is induced in the liquid that can be detected using a probe laser directed in a normal direction to the monochromatic light into the liquid. The laser beam is then deflected by the refractive index gradient, which a position-sensitive photodetector can measure. The signal detected by the photodetector is directly proportional to the amount of absorbed light and, therefore, to the sample’s absorptance given by *A* = *αd* (in the weak absorption limit). Using this method, absorptances down to 10^–4^ can be measured, and the absorption coefficient can be determined if the thickness of the sample is known. PDS has been used to detect defects in inorganic semiconductors and CT states in organic donor/acceptor blend semiconductors.^1,10,22,29^

Fourier transform photocurrent spectroscopy (FTPS) and sensitive EQE measurement are two techniques that can be used to detect subgap absorption features via photocurrent measurements. Both techniques are based on measuring the photovoltaic EQE (i.e., the ratio of photogenerated and collected electrons to incident photons), assumed to be directly proportional to the absorption coefficient in the weak absorption limit (EQE ∝ *αd* ≪ 1). In FTPS, a Fourier transform infrared (FTIR) spectrometer, which is an interferometer, is used as a light source (see Figure 1b).^30^ The FTIR spectrometer output is white light within which each wavelength is modulated at a specific frequency. This light is then used to excite the device under test (DUT), which has either a structure of a solar cell (active layer sandwiched between two electrodes) or a lateral device structure (an active layer with two coplanar electrodes). The photocurrent of the DUT is then measured as a function of wavelength. The magnitude of the photocurrent signal is then translated into an EQE signal which can be correlated with the absorption at each wavelength, assuming the internal quantum (IQE) is spectrally flat. In sensitive EQE measurements, similar to FTPS, the photocurrent at each wavelength is used to obtain an EQE as a proxy for absorption, with the difference that monochromatic light is used as the pump and the photocurrent is detected by a lock-in amplifier.^31^ Using these measurement techniques, absorptances down to 10^–6^ have been successfully measured. FTPS and sensitive EQE measurement have been used to detect trap states in inorganic semiconductors and CT state absorption features in organic solar cells.^19,32−34^

Recently, an ultrasensitive EQE measurement setup was introduced by Zeiske et al.^35,36^ A schematic of the setup is shown in Figure 1c. In this measurement, a high-performance commercial PerkinElmer spectrophotometer (Lambda 950) is used as the light source providing an extended wavelength regime from 175 nm up to 3300 nm. This double monochromator light source provides minimum stray light and undesired harmonics in the pump beam, which can be mistaken for subgap signals. The output light of the monochromator is directed to a multiblade optical chopper (OC) wheel, which physically chops the probe light at ω = 273 Hz. The chopped light beam is then focused onto the DUT. The photocurrent of the DUT is detected with a lock-in amplifier followed by passing through a low-noise precurrent amplifier with variable gain.

The dynamic range is given by DR = −10 log_10_(EQE_NE_), where EQE_NE_ is the noise equivalent EQE signal. Once an appropriate light source has been engineered in this way, the sensitivity of the EQE measurement is determined by the electrical noise. Notably, with typical monochromators providing output powers on the order of μW or less, photocurrents from nanoamps down to femtoamps can be measured in an ultrasensitive EQE experiment.

When measuring sensitive EQE (in the absence of white light bias), the total electrical noise can be written as the sum of the powers of all noise components, namely, thermal noise, electrical shot noise, hum noise, microphonic, and preamplifier noise. A detailed description for each of these noise components and how to minimize them is given in the Supporting Information. Ultimately, an absorptance down to 10^–10^ can be detected by this measurement setup, corresponding to a dynamic range of 100 dB.

## The Problem of Optical Cavity Interference

It is often assumed that α of the active layer in the weak absorbance limit follows the EQE spectrum via EQE(*E*) ∝ 2α(*E*)*d*, where *d* is the junction thickness, and the factor of 2 is introduced to account for the double pass of the beam due to the reflective electrode (this also holds for the FTPS, when performed on a solar cell). However, the EQE spectra in organic solar cell structures are also influenced by the optical interference (between incoming and reflected photons) due to the low finesse cavity effect imposed by the reflective top electrode. The cavity effect generally distorts the spectral shape of the EQE, experimentally seen as thickness-dependent features in the EQE. Accounting for interference, the EQE in the *αd* ≪ 1 limit can be expressed as^36^
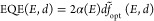
1where *f̃*_opt_ (*E*) is an energy- and thickness-dependent function determined by the governing wave optics in the thin-film device structure. Figure S2 demonstrates how the special line-shape of the EQE varies with junction thickness due to the thickness dependence of the function *f̃*_opt_ (*E,d*). To reliably determine α, it is important to correct the EQE spectral shapes for cavity-induced distortions.^37^

## Main Absorption Features in Organic Semiconductors and Their Blends

The processes of light absorption, charge generation, and recombination in organic semiconductors and their blends typically involve several species and states, namely, singlet excitons, CT states, trap states, and free charges. In particular, the generation and recombination of free charges from photons generally take place via Coulombically bound electron and hole states such as excitons and/or CT states. Therefore, free charges do not appear directly in the subgap absorption spectrum. Conversely, optical transitions associated with excitons, CT states, and midgap traps are reflected in the subgap absorption from which these states can be parametrized.

**Figure 2:**
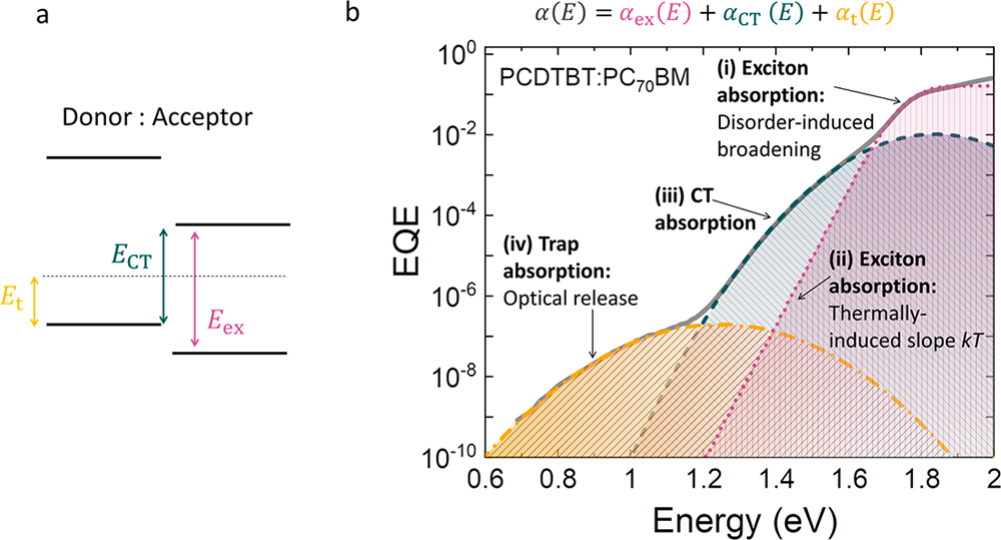
(a) Schematic energy level diagram at the donor–acceptor interface including exciton (*E*_ex_), CT (*E*_CT_), and midgap trap (*E*_t_) states. (b) The EQE spectrum of an exemplary material system PCDTBT:PC_70_BM. Near the absorption onset (∼1.8 eV) the broadness of the onset is governed by excitonic energetic disorder (region i). At lower energies, CT states dominate (region iii). An excitonic absorption tail with slope *kT* (region ii) would have been expected in the absence of the dominance of the CT states in that spectral region. A Gaussian absorption feature can be detected at the lowest energies due to the midgap trap states (region iv). We note that this qualification is in the absence of any perturbing interference features.

Figure 2 demonstrates the contributions of singlet excitons, CT states, and midgap trap states in the subgap absorption of a typical organic photovoltaic device with a fullerene-based acceptor, using a PCDTBT:PC_70_BM blend as a model system. The total absorption coefficient versus energy, α(*E*), can be written as a superposition of the absorption coefficients due to singlet excitons, CT states, and midgap traps (see Figure 2a):
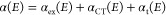
2

These absorption features, if spectrally deconvoluted, can be used to parametrize singlet excitons, CT, and trap states. Whether this is possible or not depends on the relative energies of the states. In general, however, the EQE (or rather absorption) spectrum can be divided into four different regions as indicated in Figure 2b. In the following section, we will explain each of these different regions and components of the subgap absorption and their relation to photovoltaic device performance metrics.

## Subgap Absorption Due to Excitons (Regions i and ii)

In the presence of electron–phonon scattering, or static lattice distortions, crystalline semiconductors exhibit subgap light absorption near their onset. Therefore, the spectral shape of the absorption is broader than predicted by the dispersion of the bands of a hypothetical perfect crystal.^38^ The subgap absorption in the realistic case, regardless of the purity, commonly displays an exponential tail below the bands and follows a form which is known as the Urbach rule:
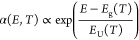
3where *E*_g_ is the bandgap energy, and *E*_U_ is the Urbach energy. In inorganic semiconductor systems it has been empirically suggested that the Urbach energy is composed of a dynamic and a static disorder component^39−41^ so that *E*_U_(*T*) = *E*_U,D_(*T*) + *E*_U,S_.

The Urbach energy has been frequently used as a measure of energetic disorder in organic semiconductors and their blends.^16,17,42−48^ However, directly fitting the subgap absorption tail with eq 3 in these systems is questionable. This is because, as shown in Figure 2b, the subgap absorption features in organic semiconductors are not always exponential. Furthermore, due to the experimental limitations of PDS and EQE measurements, a plot such as the one shown in Figure 2b is often obtained with a very limited dynamic range below the absorption onset. Therefore, an exponential fit may not be reliable due to the possible contribution of the absorption tail with additional nonexponential contributions from CT and trap states. This may ultimately result in an arbitrary and apparently energy-dependent *E*_U_.

**Figure 3:**
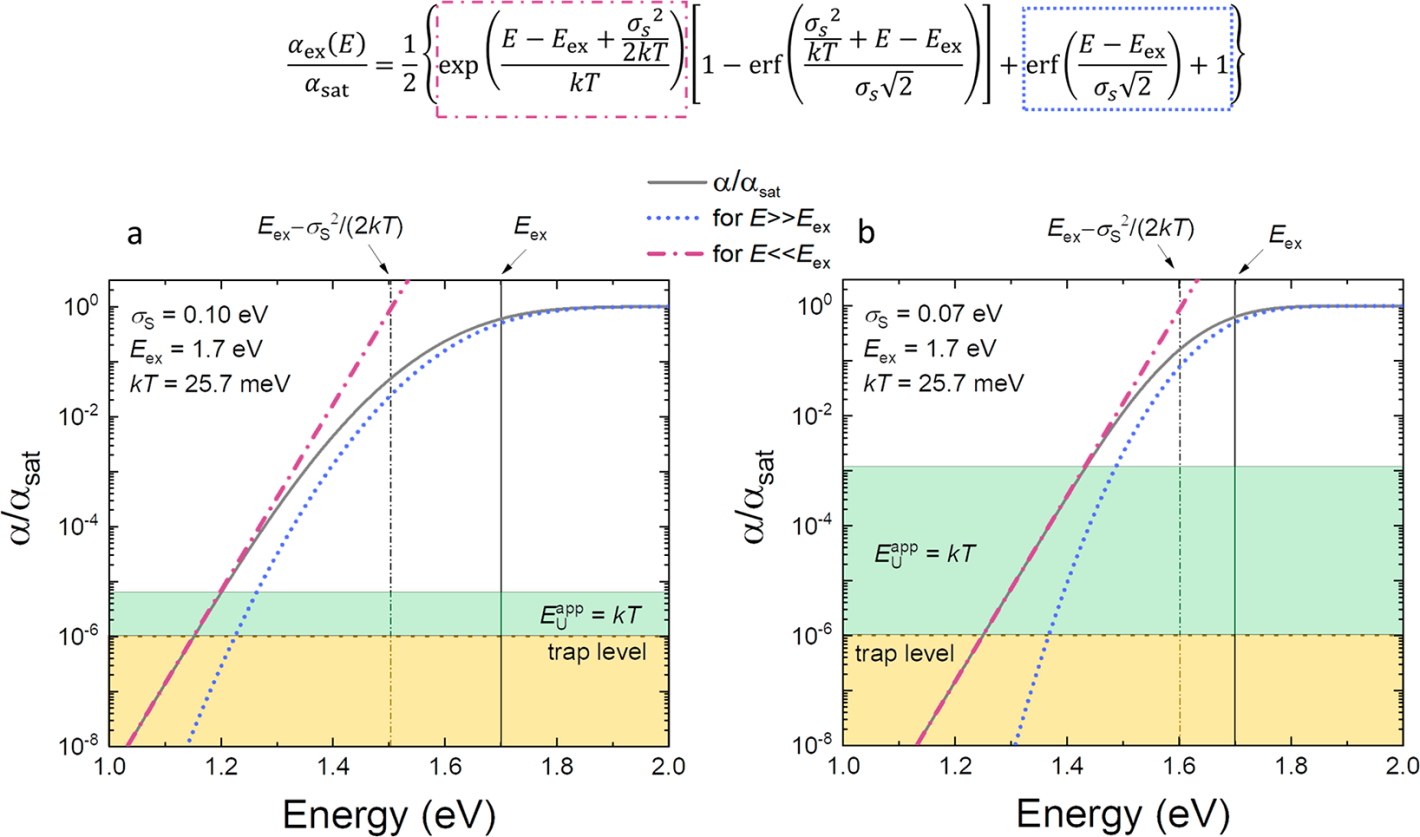
Normalized α for a Gaussian DOS is plotted for (a) σ_*s*_ = 0.10 eV and (b) σ_s_ = 0.07 eV at room temperature. In both a and b the purple and blue colors indicate α in the limit of *E* ≪ *E*_ex_ and *E* ≫ *E*_ex_, respectively. For *E* ≪ *E*_ex_, α converges to an exponential function with a slope described by *E*_U_^app^ = *kT* (green region). For *E* ≫ *E*_ex_, α converges to 1 if normalized to α_sat_. The larger σ_s_, the more sensitive the EQE measurement must be to show that *E*_U_^app^ = *kT* at low energies. Note that deep trap state absorption typically dominates for α/α_sat_ below 10^–6^, which narrows the range of exponential decay where *E*_U_^app^ = *kT* (especially for large σ_*s*_).

For a more reliable evaluation of the Urbach energy, the inverse logarithmic slope of the absorption coefficient in the subgap region may instead be used:^18^
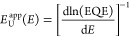
4assuming EQE(*E*) ∝ α(*E*). Here, we superscripted *E*_U_ with “app” to indicate that eq 4 results in an apparent energy-dependent Urbach energy, which should be ideally constant within a would-be exponential range. Combined with US-EQE measurements (described in the previous section), Kaiser et al.^18^ recently observed that the exciton subgap absorption at energies much below the optical gap in organic semiconductors is described by Urbach energies equivalent to the thermal energy *kT* (see Figure S3). We note that a similar finding was initially observed by Franz Urbach in silver halides.^38^ Importantly, the Urbach slope for excitons in molecular solids does not appear to depend on static disorder at energies well-below the optical gap. At photon energies closer to the optical gap though, the broadening of the subgap line shape is dominated by the energetic disorder. This provides a means to determine energetic disorder associated with the distribution of molecular orbitals as recently proposed by Kay et al.^15^ In this study, a Gaussian density of states^49^ is assigned to the excitonic states in conjunction with Boltzmann-like occupation probability (assuming that the excitonic states are more delocalized compared to the ground state). It was found that the subgap absorption coefficient due to excitons follows
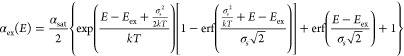
5where α_sat_ is a prefactor and σ_*s*_ is the energetic static disorder. Here, *E*_ex_ denotes the mean exciton energy associated with the 0–0 transition between the ground state and first excited singlet state of the chromophores. For blends, this will be dominated by the excitons in the component having a smaller optical gap. Hence, *E*_ex_ = min(*E*_D_, *E*_A_), where *E*_D_ and *E*_A_ is the corresponding exciton energy of the donor and acceptor component, respectively. In Figure 3, normalized α_ex_ is plotted for *E*_ex_ = 1.7 eV and two different values of σ_*s*_. As shown, the first term of eq 5 is responsible for the exponential region and the last term describes the broadness of the absorption onset which is defined by the energetic disorder. This model explains the broadened absorption onset of organic semiconductors. The effective optical gap (*E*_opt_), as observed experimentally via absorption spectroscopy, is red-shifted with respect to exciton energy so that .

Equation 5 can be used to characterize the subgap absorption due to the singlet excitons in the spectral subgap region where the contributions of CT and trap states are absent or negligible. From such parametrization, the excitonic energetic disorder can be obtained.

## Subgap Absorption Due to Charge-Transfer States (Region iii)

CT state absorption is believed to be induced by intermolecular donor–acceptor transitions. These transitions are typically described by nonadiabatic Marcus theory,^50,51^ in which the CT state is treated as a molecular state. In this picture, the absorption of a single CT state is defined by the energy difference of the ground state and the excited state *E*_CT_ and the reorganization energy λ_CT_ associated with these transitions. The corresponding α_CT_(*E*) spectral line shape is generally expected to take the Gaussian shape of the form
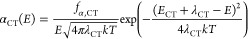
6where the prefactor *f*_α_ is related to the density of the CT states (*N*_CT_) and the associated oscillator strength (*f*_σ_) via *f*_α,CT_ = *N*_CT_*f*_σ,CT_. Equation 6 is often applied to fit spectra obtained from subgap region EQE measurements to parametrize CT states.^20,52−58^ As explained earlier, it is important to note that the spectral shape of the EQE does not always follow α(*E*) due to the distortions caused by optical interference effects. While such distortions may appear minor when plotting the EQE on a logarithmic scale, these have been demonstrated to cause significant relative errors in the extracted *E*_CT_ and λ_CT_ values, being as large as 90%.^36^

**Figure 4:**
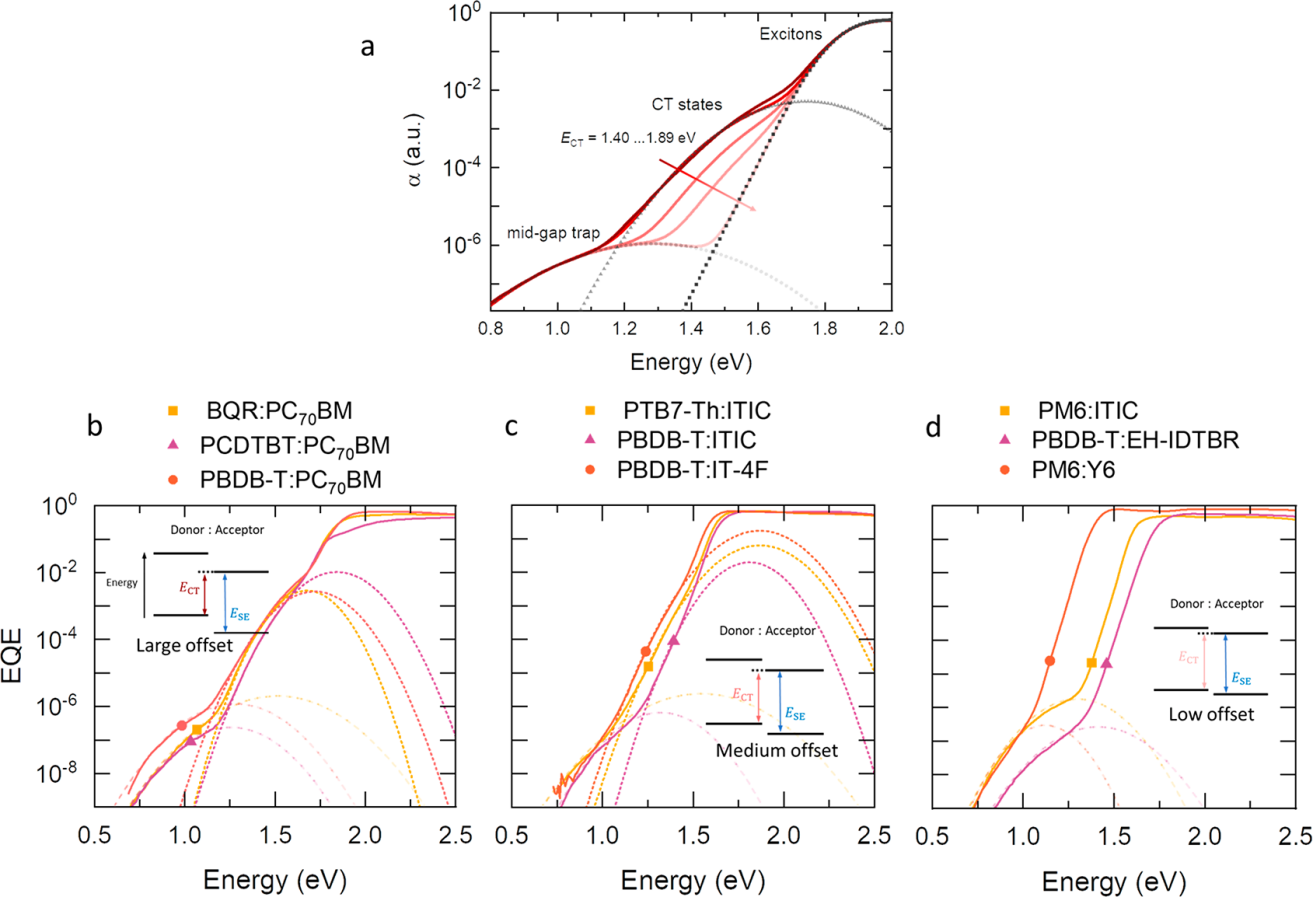
(a) US-EQE of hypothetical systems in which the CT state energy is gradually increased due to reducing the offset. This results in an apparent vanishing of the CT states as their energy level approaches singlet excitons. (b-d) show experimentally measured US-EQE of some technologically relevant blends. Notably, in the low offset systems, the signature of the CT states disappears while the midgap trap states remain.

Depending on the energy offset between the donor and the acceptor, the energy of the CT states varies, and their absorption line shape appears at different energies. In Figure 4a the effect of varying the CT state energy on the absorption coefficient is emulated, assuming α_CT_(*E*) to be given by eq 6. As *E*_CT_ increases, and the energy offset between CT states and the singlet excitons of the component with the smaller energy gap (donor or acceptor) decreases, the absorption feature of the CT states eventually disappears, becoming overshadowed by the excitonic feature. This is also exemplified experimentally, as shown in Figure 4b–d. In polymer/fullerene systems such as those shown in panel b, the CT state absorption at about 1.5 eV is pronounced. In low-offset blends, including recent highly efficient organic semiconductor blends such as PM6:Y6, as shown in panel d, in turn, the excitonic absorption features are dominant, overshadowing CT state absorption. In addition, it has also been suggested that the strong coupling between the CT state and the excitons in the component with the lowest gap creates an exciton–CT hybridized state in these low offset blends.^59−63^ As a result, absorption features due to excitons and CT states become spectrally convoluted.

CT state parametrization based on the classical Marcus model (eq 6) of subgap absorption spectra has played a crucial role in our current understanding of organic donor/acceptor blends. In recent years more sophisticated models, including Gaussian static disorder^64−68^ and multiple vibration models,^69−71^ were introduced to characterize the static and dynamic disorder of CT states. In these studies, electroluminescence (EL) measurements are mainly employed since, contrary to absorption, the EL spectra of the donor/acceptor blends are dominated by the CT state emission.

## Subgap Absorption Due to Midgap Traps (Region iv)

An additional subgap feature with characteristic energy far below the effective energy gap (be it the energy of CT or exciton states) has been demonstrated in several organic semiconductors.^24,25^ Analogous to CT states, the Marcus formalism has proven ample to describe these low-energy absorption features with corresponding energy, *E*_t_, and reorganization energy, λ_t_, associated with these transitions. Zarrabi et al. have shown that the energy of these subgap states is approximately half the energy of the CT states (see Figure S4).^25^ These observations are consistent with the presence of partially radiative midgap trap states which partake in the charge generation process. The corresponding small, but detectable spectral signature in the EQE spectrum is typically 6 orders of magnitude smaller than above-gap band-to-band transitions.

**Figure 5:**
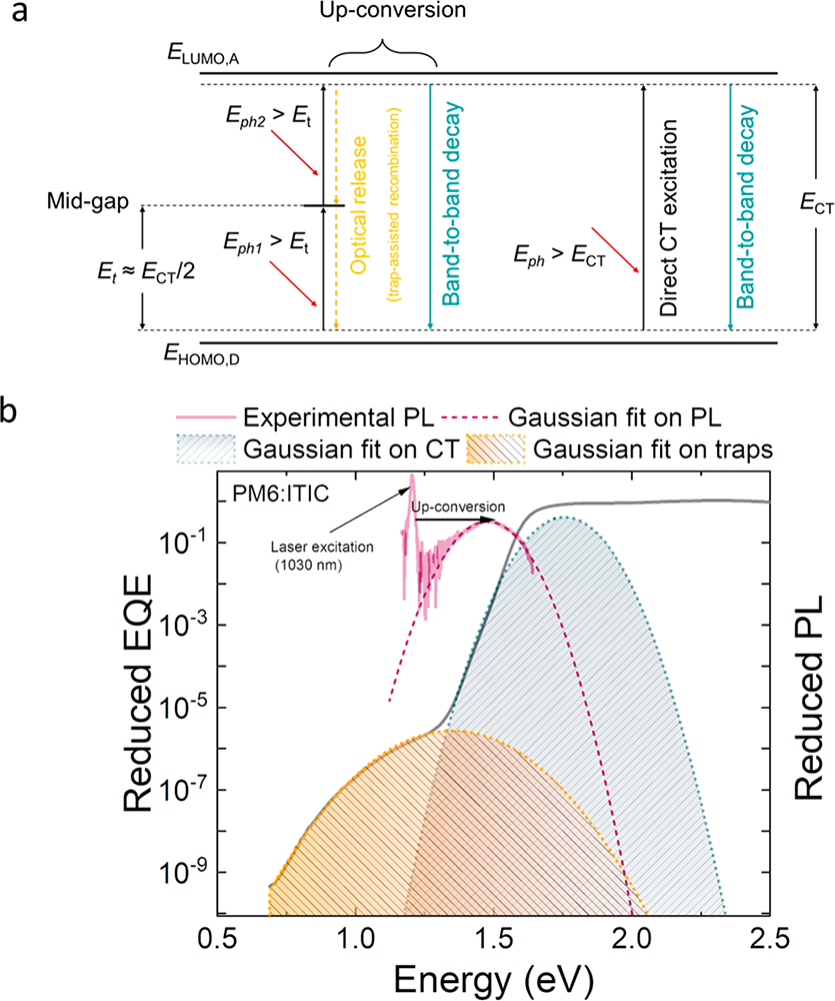
(a) Schematic of charge generation (upward arrows) and recombination (downward arrows) processes at the donor–acceptor interface including midgap trap states. (b) Reduced EQE of a PM6:ITIC device is plotted on the left axis. On the right axis the reduced PL of the same material system, excited at 1.2 eV (1030 nm), is plotted. The PL of the excited low-energy trap states emit at higher energies, consistent with optical release and subsequent photon up-conversion.

Charge generation via midgap trap states may occur through a two-step optical release process in which, on average, two photons (with energies within *E*_t_ < *E* < *E*_CT_) are needed to complete the generation of a free electron–hole pair (see Figure 5a). In this process a free hole is generated as the result of an optical excitation and subsequent charge transfer of an electron from the highest occupied molecular orbital of the donor (E_HOMO,D_) to a trap state, while a free electron is generated following the optical excitation of a trapped electron to the lowest unoccupied molecular orbital of the acceptor (E_LUMO,A_), thus contributing to the photocurrent. This nonlinear process has further been confirmed by the observation of up-conversion in the photoluminescence. In Figure 5b the left axis is the reduced EQE (EQE times energy *E*) plotted versus photon energy for PM6:ITIC. The absorption features of CT states and trap states, and the associated Gaussian fitting, are shown. The reduced PL (PL divided by energy *E*) at a pump wavelength of 1030 nm is shown on the right axis. Since this photon energy is not sufficient to excite CT or exciton states, it will only induce transitions associated with trap states. The subsequent PL of these excited states, however, shows emission at higher energies and is consistent with emission from CT states, which confirms the presence of optical release from trap states and the resulting up-conversion into CT states.

## Organic Semiconductor Photovoltaic Device Metrics: The Radiative Limit of Open-Circuit Voltage

Subgap absorption and the shape of the EQE below the gap have important implications for the device performance, most notably the open-circuit voltage. As shown by Rau,^72^ the generalized Planck equation proposed by Würfel,^73^ which relates emission and absorption of photons in semiconductors, can be extended to the electroluminescence (EL) and the photovoltaic action in solar cells. In accordance with Rau’s reciprocity relation, for a given photon energy *E* and applied voltage *V*, the photovoltaic EQE is related to the electroluminescent flux (ϕ_EL_) via
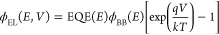
7for *E* ≫ *kT*. Here, ϕ_BB_(*E*) is the blackbody spectral photon flux of the surrounding environment (at *T* = 300 K) and given by
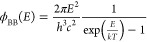
8where *h* is the Planck constant and *c* the speed of light.

Based on these considerations, the open-circuit voltage of a solar cell can generally be expressed as
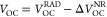
9where  is the open-circuit voltage loss caused by nonradiative processes which only depends on the radiative quantum efficiency η_EL_ for EL, and *V*_OC_^RAD^ is the radiative limit of the open-circuit voltage given by
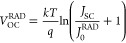
10corresponding to the upper limit of *V*_OC_ expected in the absence of nonradiative losses. Here, *J*_SC_ = *q*∫_0_^∞^EQE(*E*) ϕ_sun_(*E*) d*E* is the short-circuit current density (ϕ_sun_(*E*) is the incident spectral photon flux of the sun), while
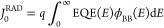
11is the expected radiative dark saturation current density. While *J*_SC_ is dominated by above-gap EQE, *J*_0_^RAD^ is in turn critically dependent on the onset of the EQE and the shape of the subgap EQE (because ϕ_BB_(*E*) increases exponentially with decreasing *E* in the energy region of interest i.e., *E* ≫ *kT*). As a result, subgap features generally correlate with decreased *V*_OC_^RAD^ and increased radiative open-circuit voltage losses.

**Figure 6:**
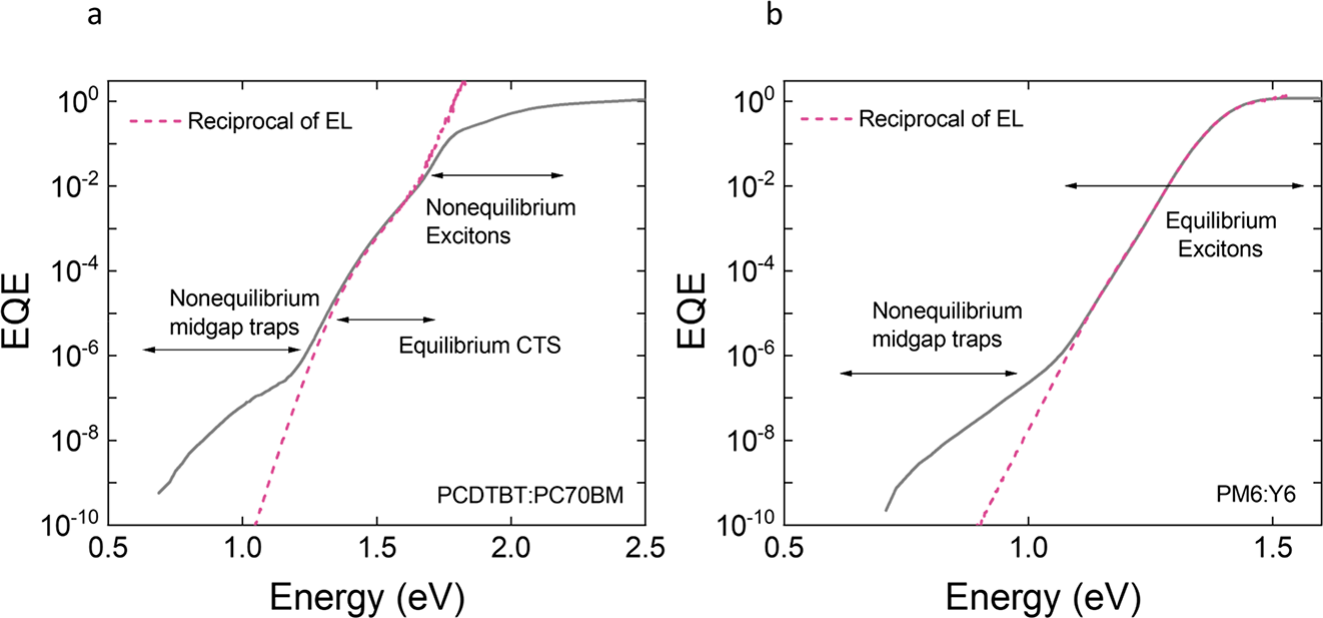
(a) The EQE of a high offset system (PCDTBT:PC_70_BM) and the reciprocal EL spectrum are shown. The reciprocity between EQE and EL is valid only for the CT states which are in chemical equilibrium with charge-separated (CS) states. (b) The EQE of a low offset system (PM6:Y6) and the reciprocal EL spectrum are shown. The reciprocity between EQE and EL is valid for the excitons which are in chemical equilibrium with CS states.

An essential assumption behind eq 7, and by extension eq 9, is that the chemical potential of the emissive species is equal to the chemical potential (i.e., the quasi-Fermi level splitting) of the free charges, or, in other words, that they are in a chemical equilibrium with each other. Figure 6 shows the EQEs expected from the EL at forward bias comparable to the open-circuit voltage, compared to the measured EQEs for systems with large and small offsets. From Figure 6a, it can be seen that for high-offset systems (exemplified by PCDTBT:PCBM) the reciprocity between absorption and electroluminescence, along with eq 11, only holds for CT states. Deviations are seen for both midgap states and excitons, suggesting that these states are not in equilibrium with free charge carriers under open-circuit conditions. As a result, we generally expect *J*_0_^RAD^ ∝ exp(−*E*_CT_/*kT*) and *V*_OC_^RAD^ ∝ *E*_CT_/*q* (under one sun illumination) in high-offset donor–acceptor systems,^20^ consistent with a large body of experimental evidence from fullerene-based acceptor BHJs.^52,65,74−79^

On the other hand, in low-offset systems (such as PM6:Y6), shown in Figure 6b, the excitonic region of EQE agrees with the reciprocal of the EL, suggesting that excitons are also in chemical equilibrium with free electron-hole pairs under open-circuit conditions (at 1 sun) in this case. This is consistent with recent findings proposing that excitons are in chemical equilibrium with free charges in low-offset systems (see ref (80) and references therein). Provided that the above-gap and subgap EQE tail is dominated by excitons, we then expect *V*_OC_^RAD^ = *V*_OC,above-gap_^RAD^ – Δ*V*_OC,sub-gap_^RAD^. Here, *V*_OC,above-gap_^RAD^ corresponds to the idealized radiative limit expected in the absence of subgap absorption, where *V*_OC,above-gap_^RAD^ ∝ *E*_ex_/*q* under one sun illumination. However, because of the exponential subgap absorption tail (see eq 5), an additional radiative open-circuit voltage loss Δ*V*_OC,sub-gap_^RAD^ will be present, which depends on the static disorder σ_*s*_ of the excitons; for small σ_*s*_, the subgap tail induced voltage loss can be approximated as^18^
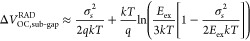
12

**Figure 7:**
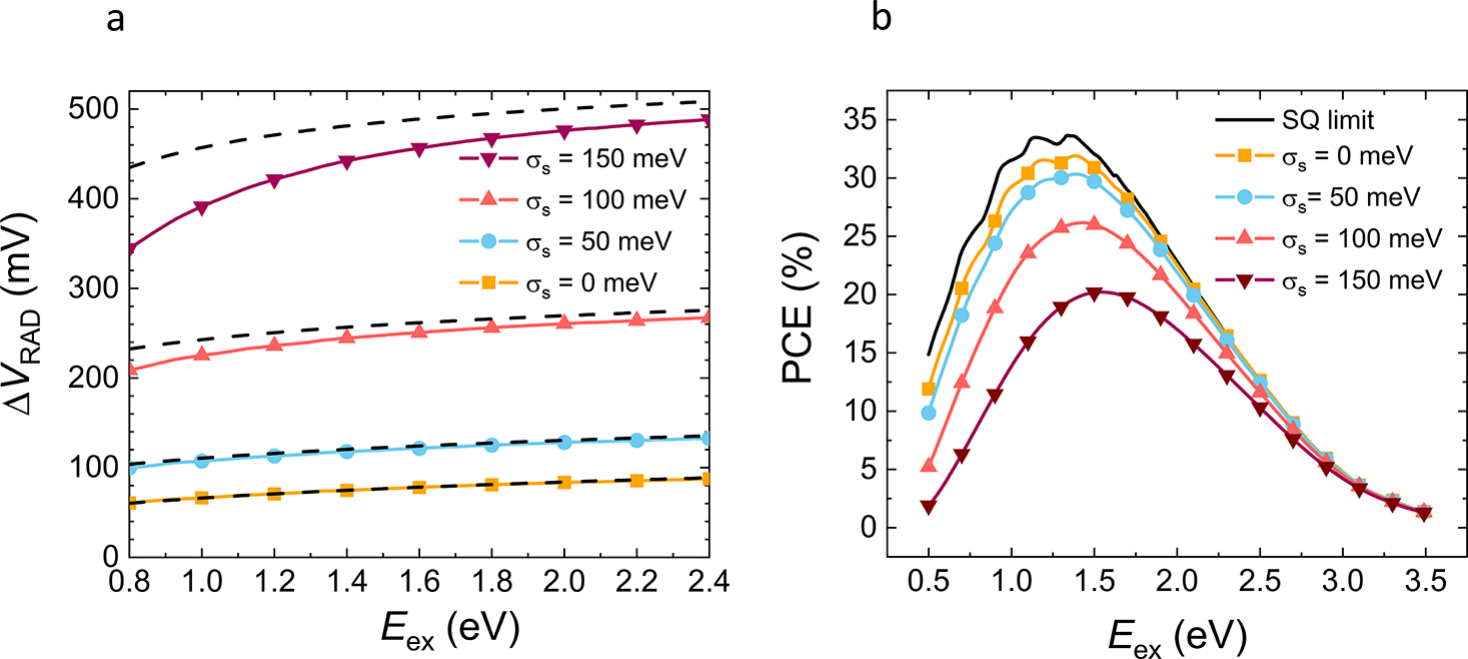
(a) The radiative voltage loss Δ*V*_OC_^RAD^ as a function of the exciton energy for varying degree of Gaussian static disorder σ_*s*_ is plotted as per eq 5 (solid lines with symbols). The dashed lines correspond to analytical approximation in accordance with eq 12. (b) The corresponding radiative PCE limit based on eq 5, assuming EQE = α_ex_/α_sat_ and ideal charge collection, is shown (solid lines with symbols) for varying σ_*s*_. The SQ limit, representing the ideal case with no subgap absorption, is included for comparison (indicated by the black solid line). An unavoidable radiative voltage loss relative to the SQ limit is present due to the thermal broadening. The PCE decreases with respect to the SQ limit as σ_*s*_ increases.

Hence, even in the absence of excitonic disorder (σ_*s*_ = 0), a subgap voltage loss induced by the *kT* tail remains, reducing the optimal PCE of organic solar cells.^18^Figure 7a,b shows the corresponding radiative limits for *V*_OC,sub-gap_^RAD^ and PCE as a function of *E*_ex_ for different σ_*s*_, and assuming EQE = α_ex_/α_sat_ as per eq 5. The idealized case without subgap absorption (*V*_OC_^RAD^ = *V*_OC,above-gap_^RAD^), corresponding to the Shockley–Queisser (SQ) limit for a gap given by *E*_ex_, has been included for comparison.

Finally, a deviation from eq 7 is generally expected for absorption features associated with midgap trap states at voltages comparable to the *V*_OC_ in organic solar cells. This can be attributed to the different voltage-dependence expected for EL from traps, being of the form , where *n*_rad_ is the so-called radiative ideality factor.^81−83^ Accounting for the presence of radiative midgap states, characterized by *n*_rad_ = 2, eq 11 can be extended as^25^
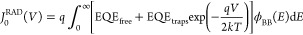
13where EQE = EQE_free_ + EQE_traps_ with EQE_free_ corresponding to the deconvoluted EQE contribution from CT states and/or excitons and EQE_traps_ the EQE contribution from charge carrier generation via deep traps. Consequently, eq 11 is not generally valid in the presence of radiative midgap trap states, which make *J*_0_^RAD^ voltage-dependent as shown in eq 13. In the limit of *V* → 0, eq 13 reduces to eq 11. Under one sun conditions, at open-circuit (*V* = *V*_OC_), the midgap trap contribution to the radiative current may be neglected, and *J*_0_^RAD^ is dominated by the subgap EQE associated with free charge carriers. At lower light intensity conditions relevant for photovoltaic indoor applications and organic photodetectors, however, the radiative open-circuit voltage losses from midgap states may become important.^25,84^ It has been shown that the noise current of organic photodetectors is determined by the midgap trap states rather than the bandgap.^84^ Such limitation has resulted in specific detectivities several orders of magnitude smaller than the expected thermodynamic limits ignoring the midgap traps. Appreciating and understanding the midgap trap states is crucial for improvement of specific detectivities via mitigating the (apparently) inevitable midgap trap states which most likely originate from unavoidable imputirity levels set by the limitations of wet chemistry purification methods.^84^

In this Perspective, the established experimental techniques used to measure the subgap absorption of semiconductors, including PDS, FTPS, and ultrasensitive EQE measurements, have been reviewed, their dynamic ranges have been compared, and the amount of information each has historically revealed has been briefly discussed. We have shown that the ultrasensitive EQE measurement has the highest dynamic range, and it can detect the absorptance down to 10^–10^. We then more specifically discussed the importance of the subgap absorption in organic semiconductor donor/acceptor blends. We have shown that various subgap absorption features corresponding to different species can be detected in the spectral line shape of the subgap absorption of organic semiconductor blends. Their spectral fingerprints appear in different regions depending on their relative energy, and they can reveal information about the static disorder, Urbach energy tail states, CT states, and trap states. To demonstrate the relevance of the analysis, several material systems were exemplified as models of how to extract this information accurately. Furthermore, we discussed the influence of the subgap species on the performance metric of organic photovoltaic devices, most notably the *V*_OC_ losses and, by extension, the power conversion efficiency. While the majority of the discussion in this Perspective has focused on solar cells, recent important insights have shown how subgap dynamics control the performance of other photovoltaic devices such as photodetectors—notably the impact of midgap trap states in limiting dark current.

## Supporting Information Available

Chemical definition, additional experimental details about sensitive EQE measurement, and Figures S1–S4 (PDF)
