# Supplementary material for: Subgap Absorption in Organic Semiconductors
Source: J Phys Chem Lett. 2023 Mar 24;14(13):3174–85. doi: 10.1021/acs.jpclett.3c00021 (PMC10084470; doi:10.1021/acs.jpclett.3c00021)
Supplement: Supplementary file 1 — jz3c00021_si_001.pdf [file jz3c00021_si_001.pdf]

Supporting Information

Subgap Absorption in Organic Semiconductors

Nasim Zarrabi [1], Oskar J. Sandberg [1,], Paul Meredith [1], Ardalan Armin [1,]**

[1] Sustainable Advanced Materials (Ser-SAM), Department of Physics, Swansea University, Singleton Park, Swansea SA2 8PP, United Kingdom

Corresponding Authors

*Email: ardalan.armin@swansea.ac.uk; o.j.sandberg@swansea.ac.uk;

Chemical definitions:

PCDTBT; Poly[N-9'-heptadecanyl-2,7-carbazole-alt-5,5-(4',7'-di-2-thienyl-2',1',3'-benzothiadiazole)]

PCPDTBT; Poly[2,6-(4,4-bis-(2-ethylhexyl)-4*H*-cyclopenta [2,1-*b*;3,4-*b*′]dithiophene)-*alt*-4,7(2,1,3-benzothiadiazole)]

PTB7-Th; Poly[4,8-bis(5-(2-ethylhexyl)thiophen-2-yl)benzo[1,2-b;4,5-b']dithiophene-2,6-diyl-alt-(4-(2-ethylhexyl)-3-fluorothieno[3,4-b]thiophene-)-2-carboxylate-2-6-diyl)]

PM6; Poly[(2,6-(4,8-bis(5-(2-ethylhexyl-3-fluoro)thiophen-2-yl)-benzo[1,2-b:4,5-b']dithiophene))- alt-(5,5-(1',3'-di-2-thienyl-5',7'-bis(2-ethylhexyl)benzo[1',2'-c:4',5'-c']dithiophene-4,8-dione)]

BQR; benzodithiophene-quaterthiophene-rhodanine

PBDB-T; Poly[(2,6-(4,8-bis(5-(2-ethylhexyl)thiophen-2-yl)-benzo[1,2-b:4,5-b']dithiophene))-alt-(5,5-(1',3'-di-2-thienyl-5',7'-bis(2-ethylhexyl)benzo[1',2'-c:4',5'-c']dithiophene-4,8-dione)]

$PC_{70}BM$; [6,6]-Phenyl-C71-butyric acid methyl ester

BQR; benzodithiophene-quaterthiophene-rhodanine

IT-4F; 3,9-bis(2-methylene-((3-(1,1-dicyanomethylene)-6,7-difluoro)-indanone))-5,5,11,11-tetrakis(4-hexylphenyl)-dithieno[2,3-d:2',3'-d']-s-indaceno[1,2-b:5,6-b']dithiophene

Y6; 2,2'-[[12,13-Bis(2-butyloctyl)-12,13-dihydro-3,9-dinonylbisthieno[2",3":4',5'] thieno[2',3':4,5]pyrrolo[3,2-e:2',3'-g][2,1,3]benzothiadiazole-2,10-diyl]bis [methylidyne(5,6-chloro-3-oxo-1H-indene- 2,1(3H)-diylidene)]]bis[propanedinitrile]

ITIC; 3,9-bis(2-methylene-(3-(1,1-dicyanomethylene)-indanone))-5,5,11,11-tetrakis(4-hexylphenyl)-dithieno[2,3-d:2',3'-d']-s-indaceno[1,2-b:5,6-b']dithiophene

EH-IDTBR; (5Z)-3-ethyl-2-sulfanylidene-5-[[4-[9,9,18,18-tetrakis(2-ethylhexyl)-15-[7-[(Z)-(3-ethyl-4-oxo-2-sulfanylidene-1,3-thiazolidin-5-ylidene)methyl]-2,1,3-benzothiadiazol-4-yl]-5,14-dithiapentacyclo[10.6.0.03,10.04,8.013,17]octadeca-1(12),2,4(8),6,10,13(17),15-heptaen-6-yl]-2,1,3-benzothiadiazol-7-yl]methylidene]-1,3-thiazolidin-4-one

Note 1: How to make EQE measurements more sensitive

Considering a photovoltaic (diode) device with a negligible series resistance, the diode equation is $J = \frac{V}{R_{\mathrm{sh}}} + J_0\left[\left(e^{qV/n_{\mathrm{id}}kT}\right) - 1\right]$ where J is the total current, J_0 is the dark saturation current of the diode, V is the voltage, R_{sh} is the shunt resistance, q is the elementary charge, k is the Boltzmann constant, T is the absolute temperature and n_{id} is the diode ideality factor . The sum of noise powers gives total noise:

$$\langle i_{\mathrm{noise}}^2\rangle = \langle i_{\mathrm{thermal}}^2\rangle + \langle i_{\mathrm{shot}}^2\rangle + \langle i_{\mathrm{mic}}^2\rangle + \langle i_{\mathrm{hum}}^2\rangle + \langle i_{\mathrm{preamp}}^2\rangle$$

Thermal noise: thermal noise is present at all applied voltages and depends on the total parallel resistance of the device (R_p) so that the thermal noise power $\langle i_{\mathrm{thermal}}^2\rangle = \frac{4kT}{R_p}\Delta f$ where Δf is the electrical bandwidth set by the time constant of the lock-in amplifier. The resistance R_p is generally composed of both the geometric shunt (due to the pinholes and structural defects) and device-intrinsic contributions such as band-to-band or trap-mediated transitions: $R_p = \left(\frac{dJ}{dV}\right)^{-1} = \left[\frac{1}{R_{\mathrm{sh}}} + \frac{J_0 q}{n_{\mathrm{id}}kT}\left(e^{qV/n_{\mathrm{id}}kT}\right)\right]^{-1}$. Consequently, it is concluded that photovoltaic devices with larger shunt resistance are more suitable for a low-noise EQE measurement and due to the inverse exponential dependence of J_0 to the bandgap, devices with narrower bandgap semiconductors (larger J_0) will suffer more from thermal noise.

Shot noise: At zero volts, the shot noise component is zero. At voltages in the reverse bias, however, shot noise is given by $\langle i_{\mathrm{shot}}^2\rangle = 2qJ_0\Delta f$. Hence, narrower gap photovoltaic devices suffer more from this noise source. Ideally, the shunt current does not take part in the shot noise as it is not thermally activated; however, it has been observed that the shot noise scales with the total current (rather than J_0) so that $\langle i_{\mathrm{shot}}^2\rangle \approx 2qJ\Delta f$. The exact reason behind this observation may vary, but it is more likely because the total current passing through the DUT

is passed through the pre-amplifier electronics components involving p-n junctions. Hence the total current can be thermally activated and subject to the shot noise.

Microphonic (pick-up) noise: This noise component is due to the transduction of mechanical vibrations in the environment to electrical signals. It is, therefore, essential to ensure about mechanical stability of the DUT, wires and the pre-amplifier during the measurement.

Mains Hum noise: this noise is induced due to the 50/60 Hz alternative currents in the mains power cables and can have a dramatic effect. Mains hum not only creates significant noise peaks at 50/60 Hz and its harmonics but also increases the noise floor at low frequencies (<1000 Hz) relevant to EQE measurements. In order to avoid this source, choosing a chopping frequency (of the monochromator light) at a frequency other than the harmonic frequency is necessary but not sufficient. The DUT should be mounted in a closed metallic sample holder acting as a Faraday cage in order to reduce the hum noise. It is also important to employ low-noise coaxial cables to minimize the pick-up noise due to the wires.

Pre-amplifier noise: in order to detect small currents, it is inevitable to pre-amply the current prior to phase-sensitive detection. A high-gain pre-amplifier is required with an input noise level lower than the noise level of the DUT. In the case of measuring sensitive EQE at a nonzero voltage bias, the pre-amplifier must be equipped with an ultra-low noise voltage source, providing a constant voltage with a noise level smaller than the device's shot noise.

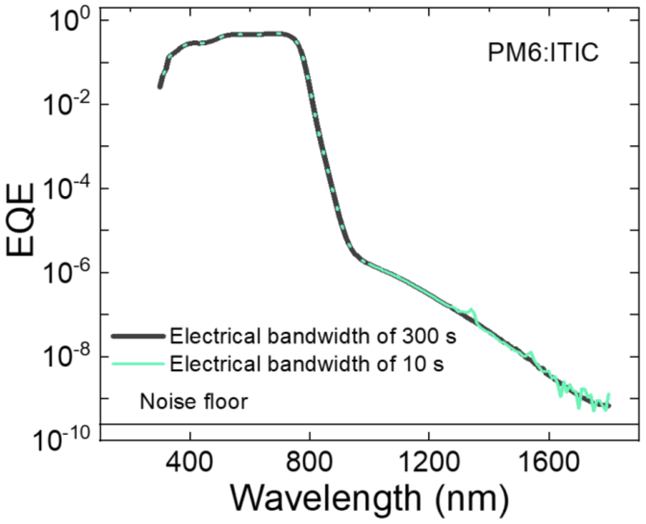

Figure S1. The signal-to-noise (SNR) ratio is improved by increasing the integration time (from 10s to 300s) of the US-EQE measurement for a typical PM6:ITIC organic solar cell.

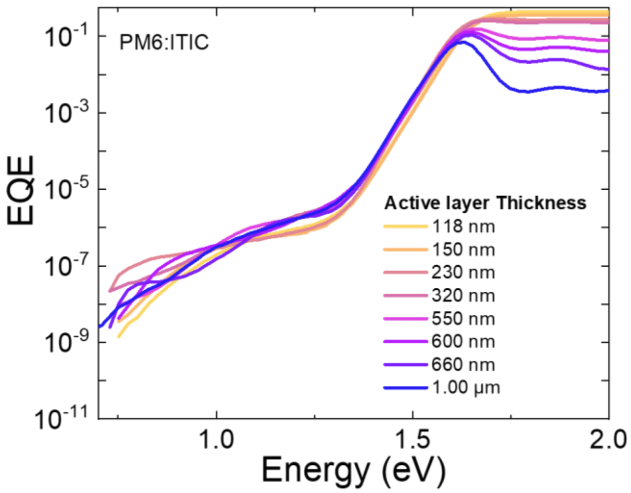

Figure S2. The effect of optical interference on the shape of the EQE in the subgap region.

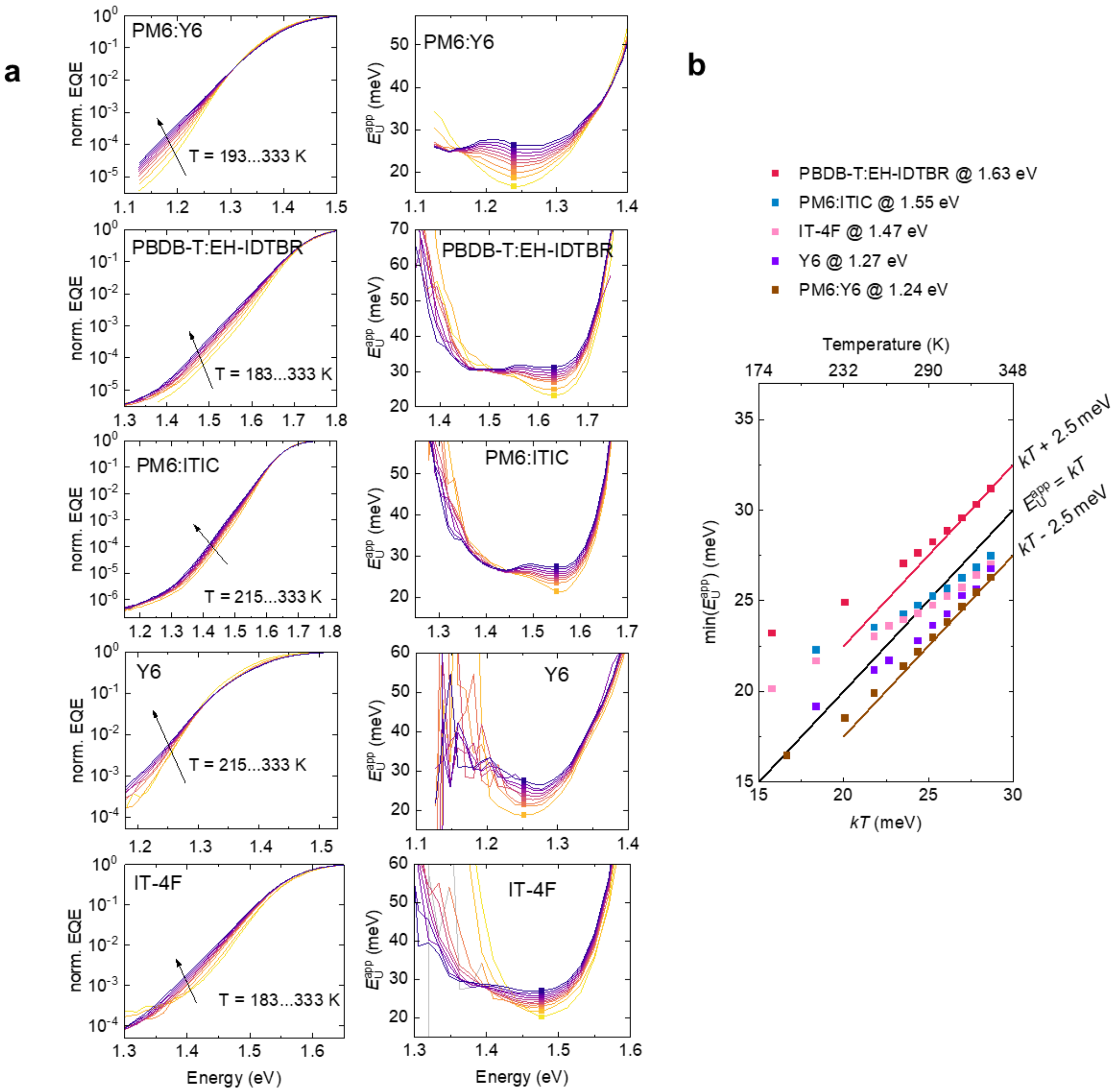

Figure S3. (a) Normalised subgap EQE and the respective apparent Urbach energy is plotted at different temperatures. (b) Extracted apparent Urbach energy for blend BHJ and neat material systems is plotted versus temperature. The $E_{\mathrm{U}}^{\mathrm{app}}$ is linear and equals kT ± 2.5 meV at higher temperatures; the offset can be attributed to interference effects. $E_{\mathrm{U}}^{\mathrm{app}}$ eventually deviates from linearity, as the spectral shape is more affected by other absorbing species at lower temperatures.

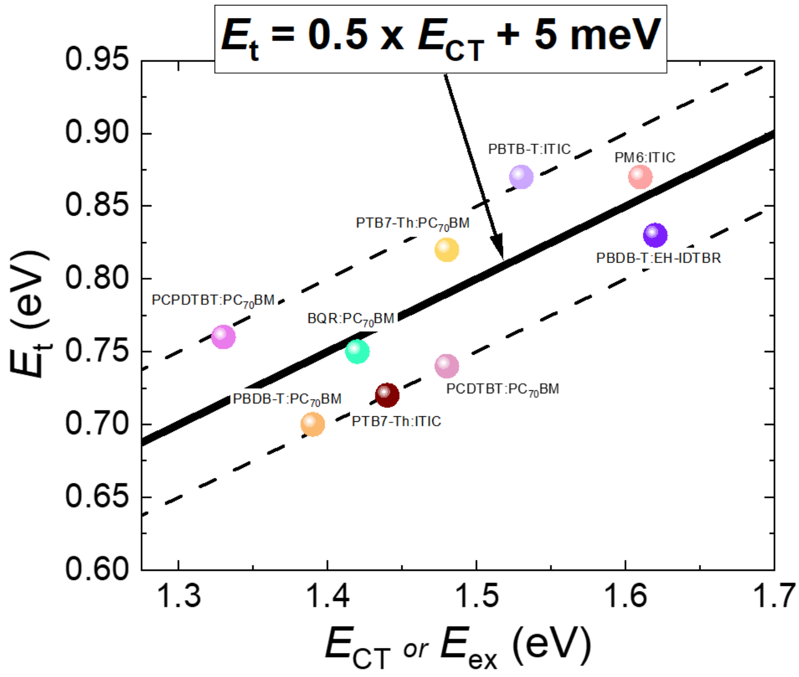

Figure S4. The corresponding trap state energy E_t extracted from Gaussian fittings to the trap state features of EQE_{PV} plotted versus the energy of the effective gap (CT state or singlet exciton) for different technologically-relevant BHJ material systems. The energy of trap states is approximately half of the energy of the CT states.
